# Supporting or complicating? The role of rods and bridges in loop stomas: a comprehensive systematic review and meta-analysis with GRADE evaluation and trial sequential analysis

**DOI:** 10.1007/s13304-025-02455-z

**Published:** 2025-12-02

**Authors:** Mohamed Abo Zeid, Kareem Khalefa, Lamees Taman, Kareem Ibraheem, Amr Alaa Azzouz Elkelany, Habiba Abdelhameed Elrefaey, Habiba Tariq Saeed, Youssef Noureldin, Amr M. Abou Elezz

**Affiliations:** 1 Faculty of Medicine, Tanta University, Tanta, Egypt; 2 Faculty of Medicine, Palestine Polytechnic University, Hebron, Palestine; 3 Southampton General Hospital UHS foundation trust, Southampton, Hampshire UK

**Keywords:** Loop stoma, Support rod, Colostomy, Ileostomy, Meta-analysis, Surgery

## Abstract

This systematic review and meta-analysis investigated the efficacy and safety of support rods and bridges in loop stoma formation, a practice frequently employed in colorectal surgery despite the associated complications and controversial efficacy. The aim of this study is to provide evidence-based guidance on their routine use. Adhering to the Cochrane Collaboration’s recommendations, we conducted this systematic review and meta-analysis systematically searching PubMed, Scopus and Web of Science for randomized controlled trials (RCTs) evaluating the efficacy of rods/ bridges in loop ileostomies. Key outcomes assessed included stoma retraction, stoma/skin necrosis, peristomal dermatitis, infection, and mucocutaneous separation. This review included six studies encompassing 1,239 patients (195 ileostomy, 1,044 colostomy). Analysis revealed no statistically significant difference in stoma retraction (RR = 0.70, 95% CI [0.37 to 1.33], *P* = 0.28). Conversely, for stoma/skin necrosis, four studies were analyzed and showed a statistically significant difference favoring the non-rod/bridge group (RR = 5.04, 95% CI [1.72 to 14.73], *P* = 0.003). For peristomal dermatitis, three studies revealed a statistically significant difference favoring the non-rod/bridge group (RR = 2.01, 95% CI [1.55 to 2.60], *P* < 0.00001), with ostomy rod use identified as a significant risk factor (OR 3.42, *p* < 0.01). Outcomes for infection and mucocutaneous separation showed no significant difference. Based on the our results, routine rod/bridge use does not appear to reduce stoma retraction and may be associated with an increased risk of necrosis and dermatitis. That is why the routine use of rod/bridge does not appear to be justified, but the small number of the included studies limit the generalizability of these results. Trial sequential analysis indicated that future high-quality RCTs with diverse and larger populations and are recommended to obtain more rigorous evidence.

**Supplementary Information:**

The online version contains supplementary material available at 10.1007/s13304-025-02455-z.

## Introduction

A Diverting loop stoma, typically a loop ileostomy or colostomy is a standard surgical practice frequently employed in elective colorectal surgery.[1, 2]. By diverting the fecal stream from the distal anastomosis, loop stomas are thought to reduce leakage-related complications and promote healing. Such practice is supported by several studies which have demonstrated the benefits of loop ileostomies in reducing the risks of anastomotic leakage and its complications [2, 3] which is a major complication of intestinal anastomoses [4] particularly in patients with low colorectal anastomosis [5, 6].

However, despite their protective role, stomas themselves are associated with significant complications affecting up to 50% of patients [7], including stoma retraction, prolapse, and ulceration [8]. Traditionally, a rod or bridge is placed under the bowel loop to prevent stoma retraction and ensure optimal fecal diversion maintaining proper postoperative positioning and reducing complications.

Although the use of support rods and bridges is widely advocated in clinical practice and recommended in surgical literature [9–12], their use remains controversial as their efficacy hasn’t been clearly established. Moreover, several studies reported higher complication rates with their use [13, 14]. While multiple randomized controlled trials have assessed the efficacy of support rods and bridges, the results remain inconsistent.

Given this clinical uncertainty and conflicting evidence, there is a clinical need for a rigorous evaluation of the available data to guide evidence based surgical decisions. Therefore, we conducted a comprehensive systematic review and meta-analysis to assess the efficacy and safety of rod or bridge use in loop stoma formation. Our aim is to provide evidence-based guidance on whether routine use of rod remains justified in modern colorectal surgical practice.

## Methods

### Study design and registration:

We conducted this systematic review and meta-analysis adhering to the PRISMA [15] (Preferred Reporting Items for Systematic Reviews and Meta-Analyses) guidelines and followed the Cochrane Collaboration’s recommendations. The GRADE approach was used to assess the strength of the evidence [16]. This study was registered on Open Science Framework (OSF) under the DOI [10.17605/OSF.IO/T5RFH].

### Eligibility criteria

To establish our inclusion criteria, we adopted the PICO framework. Studies were included if they involved patients who underwent loop ileostomy or loop colostomy and received either a rod/bridge or no supporting device. We included studies that reported at least one of the following outcomes: Stomal retraction, Stoma/Skin Necrosis, Peristomal Skin Complications, Stoma Site/Peristomal Infection, Stoma prolapse, and mucocutaneous Separation. We excluded studies with irrelevant outcomes, or unavailable complete data. Studies that included animal experiments, conference reports, reviews, retrospective studies, meta-analyses, and case reports.

### Search strategy

We conducted a systematic search through the following medical electronic databases: PubMed, Web of Science, and Scopus including studies up to 11th June 2025 using Medical Subject Headings (MeSH) targeting studies with the following keywords (“stoma” OR “ostomy” OR “ileostomy” OR “colostomy” OR “loop ileostomy” OR “loop colostomy”) AND (Rod OR bridge OR Bar OR “support rod” OR “support bridge” OR “support bar” OR “stoma rod” OR “stoma bridge” OR “stoma bar” OR “bridge device” OR “rod device” OR “bar device” OR “bridge support” OR “rod support” OR “bar support” OR “bridging rod” OR “bridging bar” OR “bridging device” OR “bridge placement” OR “rod placement” OR “bar placement”). Duplicates were removed using endnote software, then were screened based on the previously mentioned eligibility criteria through the titles and abstracts using Rayyan website [17] then the remaining articles were screened based on their full text. Any conflicts were solved by discussion.

### Quality assessment

We used the Cochrane risk-of-bias tool for randomized trials (RoB-2) [18] to assess the methodological quality of the RCTs. The tool evaluates six key aspects: (1) bias in random sequence generation, (2) bias due to deviations from intended interventions, (3) bias due to missing outcome data, (4) bias in outcome measurement, (5) bias in the selection of reported results, and (6) overall bias. Each aspect is classified as "low risk," "some concern," or “high risk”. Two authors independently conducted the risk of bias assessment with a third author resolving any disagreements.

### Data extraction

Data extraction was primarily divided into three main sections. (1) study characteristics, (2) population demographics, and (3) outcome measurements. Data from the included studies were extracted by two authors independently into an online data extraction sheet. A third author was involved in resolving any conflicts.

### Data synthesis and statistical analysis

RevMan Cochrane software [19] version 5.4, was used to analyze the data. In absence of heterogeneity a fixed effect model was used; otherwise, a random effect model was used. We analyzed dichotomous data (Stomal retraction, Stoma/Skin Necrosis, Peristomal Skin Complications, Stoma Site/Peristomal Infection, Stoma prolapse, and mucocutaneous Separation) using risk ratio (RR) with a 95% confidence interval (CI) and *P*-values < 0.05 considered statistically significant. Leave one out sensitivity analysis was conducted using OpenMeta [Analyst] software (by the Center of Evidence Based Medicine, Brown University, School of Public Health, Rhode Island State, USA).

### Assessment of heterogeneity

The chi-square test was used to assess the presence of significant heterogeneity, with *P* value of ≤ 0.1 considered statistically significant heterogeneity. In addition to I^2^ test used to evaluate heterogeneity according to I^2^ classification. I^2^ of (≤ 30%) classified as non-significant heterogeneity, (30–50%) classified as moderate heterogeneity, and (70%) classified as significant heterogeneity.

### Trial sequential analysis

To ensure the conclusiveness of our meta-analytic findings and minimize the risk of False positive (Type I error). We employed a trial Sequential analysis (TSA) using the Copen-Hagen TSA program version 0.9.5.10 Beta[20]. Parameters including a type 1 error (α) of 5%, type 2 error (β) of 20% corresponding to a statistical power of 80% were employed. We used a model variance-based heterogeneity correction. We generated both a monitoring boundaries plot and a Penalized Z-curve Plot ensuring the robustness of our conclusions for our primary outcome. As for the Monitoring boundaries plot, it employed a superiority boundary based on the O’Brien Fleming alpha spending function to mitigate type 1 errors and a futility boundary based on the O’Brien Fleming beta spending function to mitigate type 2 errors. Regarding the Penalized Z-curve Plot, the cumulative z statistic was penalized by applying the Law of Iterated Logarithm incorporating a λ value of 2. Both plots included a required information size (RIS) axis to determine whether the data is sufficient or not.

## Results

### Search results and study selection

**Fig. 1:**
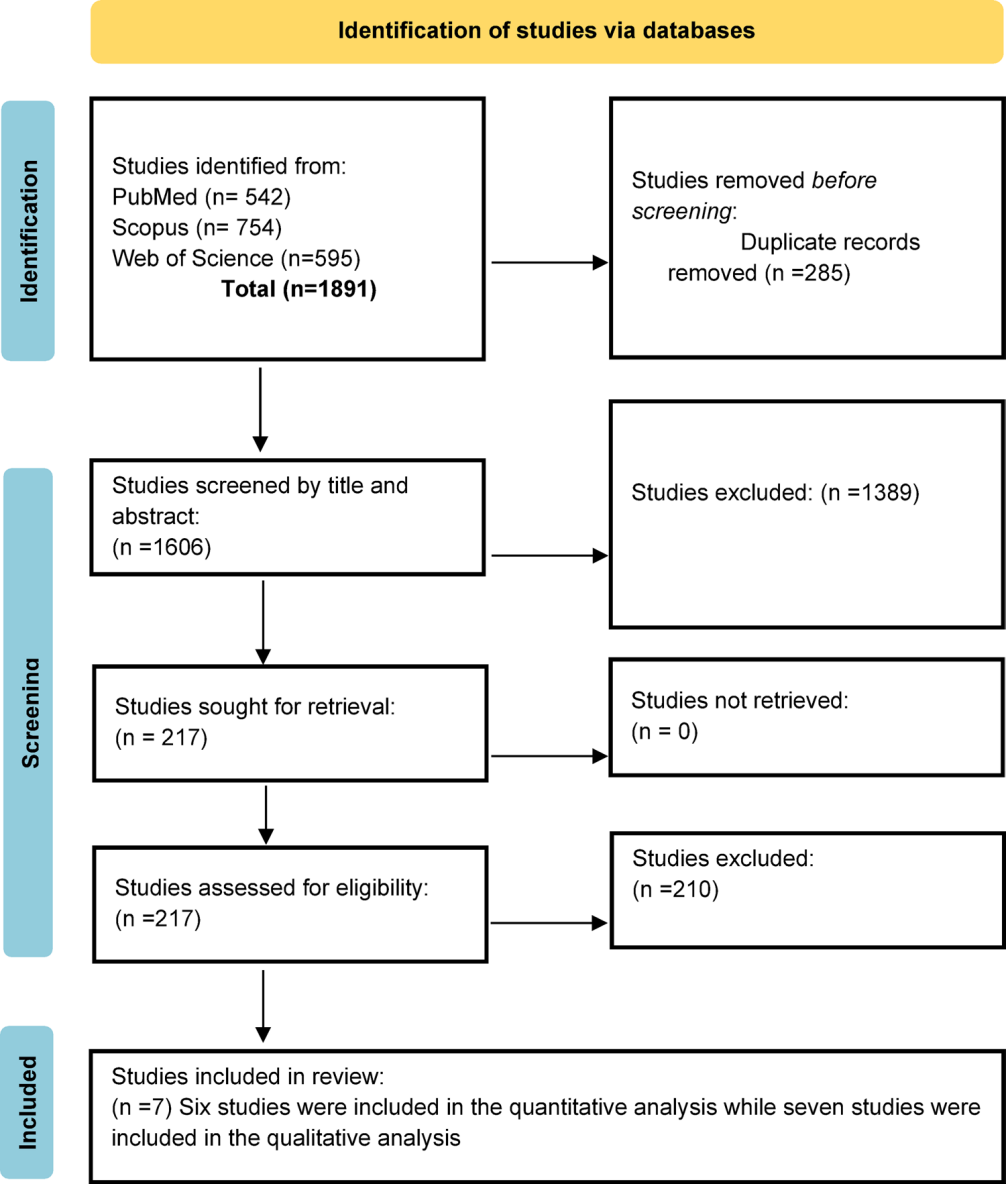
PRISMA flowchart for study selection

A total of 1891 studies were initially identified through systematic searches of PubMed (n = 542), Scopus (n = 754), and Web of Science (n = 595). 285 of which were duplicated and removed. On screening the titles and abstract of the remaining 1606 studies using the pre-defined eligibility criteria. 1389 studies were excluded for not meeting the eligibility criteria. When evaluating the full text of the remaining 217 articles, 210 studies were excluded. Only seven studies were included in the systematic review, of which six were eligible for quantitative synthesis (Fig. 1).

### Characteristics of included studies

Six studies were included in our quantitative analysis encompassing 1239 patients undergoing either ileostomy (195 patients) or loop colostomy (1044 patients). Among the included studies five studies were randomized controlled trials and one was an observational cohort study. Interventions included either rod/bridge use or no rod/bridge-less stoma. A wide range of indications were employed as rectal cancer, inflammatory bowel disease, pelvic malignancy, and perianal sepsis. Included patients mainly included adults scheduled for either elective or emergency surgeries. One study was included in the qualitative analysis (Table 1).

**Table 1:** Summary of included studies

Study ID	Study Design	Total Participants	Study arms		Type of procedure		Indication for surgery	Inclusion criteria
			Intervention	Control	Loop colostomy N (%)	Loop Ileostomy N (%)		
Zindel 2017 (Switzerland)	RCT	78	Rod	Rod-less	0	78 (100%)	1. Anterior resection 2. Reversal of Hartman’s situation 3. Ileoanal pouch formation 4. Diversion for various reasons (perianal fistula, fistula after pouch formation, pelvic sepsis)	Patients scheduled for planned protective loop ileostomy
Whiteley 2016 (Australia)	Observational cohort	515	Rod	No rod	44 (7.4%)	471 (91.4%)	1. Stratified into cancer 2. Inflammatory bowel disease 3. Diverticular disease 4. Functional disorders (intractable-incontinence-constipation) 5. Complex Perianal Sepsis 6. Intra-abdominal sepsis (perforation/anastomotic leakage) 6. Others (e.g. stoma resiting/refashioning, radiation, proctitis, familial adenomatous polyposis)	Consecutive patients older than 18 years undergoing loop ileostomy or colostomy formation were included in this study
Uchino 2017 (Japan)	RCT	308	Rod	No rod	0	308 (100%)	Cancer/dysplasia	Patients with UC who were 18 years of age or older
Franklyn 2017 (India)	RCT	151	Rod	No rod	151 (100%)	0	1. Obstructed carcinoma rectum, 2. Severe Perianal Sepsis 3. Trauma 4. Miscellaneous (pelvic malignancies, radiation proctitis, etc.)	ALL adult patients undergoing a loop colostomy (elective and emergency)
Oh 2016 (Seoul Korea)¿	Non RCT	32	Bridge	No bridge	0	32 (100%)	Except for one case in which stoma formation was performed for postoperative anastomotic leakage, all loop ileostomies were conducted on a nonemergency basis 1. Rectal cancer 2. Anastomotic leak 3. Ulcerative colitis	NA
Speirs 2006 (UK)	RCT	57	Bridge	bridge-less	0	57 (100%)	1. Ileoanal pouch formation 2. Anterior sphincter repair 3. Anterior resection 4. Colo-vesical fistula 5. Chronic constipation 6. Reversal of Hartmann’s 7. Pelvic sepsis	NA
Sabbag 2025 (France)	RCT	130	Bridge	No bridge	0	130(100%)	1. Cancer 2. Diverticulitis 3. Crohn’s disease 4. Ulcerative colitis	18 years of age or older who had an elective colorectal resection for any reason (cancer, inflammatory bowel disease, diverticulitis)

In the intervention group, 238 were female participants, the mean age was ranging from 64.3 ± 10.5 to 42.9 ± 15.2 years, while the mean BMI was ranging from 26.1 ± 4.3 to 19.5 ± 2.6 kg/m2. On the other hand, in the control group only 222 were female participants, the mean age was ranging from 64.6 ± 14.4 to 41.9 ± 15 years, while the mean BMI was ranging from 26.2 ± 4.2 to 19.8 ± 3.1 kg/m2. Reporting on operative techniques varied across studies between either open or laparoscopic approaches. ASA classification and other perioperative characteristics are shown in (Table 2).

**Table 2:** Demographics and baseline characteristics of included studies

Study ID	Study arms	N (%)	Age (years)	Sex (female) N (%)	Weight (kg)	BMI (kg/m^2^)	ASA (I/II/III)	Open surgery N (%)	Laparoscopic N (%)
Zindel 2017	Rod	44 (56.4%)	64.3 (10.5)	10 (22%)	N/A	26.1(4.3)	3/11/11	21	23 (52.3%)
	No rod	34 (43.6%)	59.3 (12.3)	12 (36%)	N/A	26.2 (4.2)	5/18/4	13	21 (61.8%)
Whiteley 2016	Rod	260 (50.5%)	59.7 (16)	106 (40.8%)	N/A	NA	N/A	N/A	N/A
	No rod	255 (49.5%)	64.6 (14.4)	105 (41.2%)	N/A	NA	N/A	N/A	N/A
Franklyn 2017	Rod	75 (49.6%)	43.0 (15.8)	33 (44%)	N/A	21.0 (3.9)	N/A	25 (33.3%)	47 (62.6%)
	No rod	76 (50.4%)	44.7 (15.3)	33 (43.4%)	N/A	20.6 (4.3)	N/A	22 (28.9%)	56 (73.6%)
Oh 2016	No bridge	12 (37.5%)	64.1 (21.5)	4 (12.5%)	N/A	21.3 (2.5)	9/2/1	9 (28.12%)	3 (9.3%)
	Short term bridge	9 (28.12%)	54.9 (22.2)	4 (12.5%)	N/A	22.6 (3.7)	8/1/0	9 (28.12%)	0
	Long term bridge	11 (34.3%)	58.4 (27.4)	2 (6.25%)	N/A	23.4 (2.1)	8/3/0	11 (34.37%)	0
Uchino 2017	Rod	154 (50%)	42.9 (15.2)	63 (41%)	N/A	19.5 (2.6)	N/A	N/A	N/A
	No rod	154 (50%)	41.9 (15.0)	48 (31%)	N/A	19.8 (3.1)	N/A	N/A	N/A
Speirs 2006	Bridge	28 (49%)	56.66 (17.96)	N/A	N/A	24.6 (3.12)	N/A	N/A	N/A
	No bridge	29 (51%)	61.66 (14.0)	N/A	N/A	24.3 (3.9)	N/A	N/A	N/A
Sabbag 2025	Bridge	67 (51.5%)	62 (10)	26 (39%)	N/A	26.0 (4.4)	4 (6.0%)/34 (51%)/29 (43%)	N/A	N/A
	No bridge	63 (48.5%)	63 (13)	24 (38%)	N/A	26.1 (4.1)	2 (3.2%)/31 (49%)/30 (48%)	N/A	N/A

### Quality assessment of the included studies

**Fig. 2:**
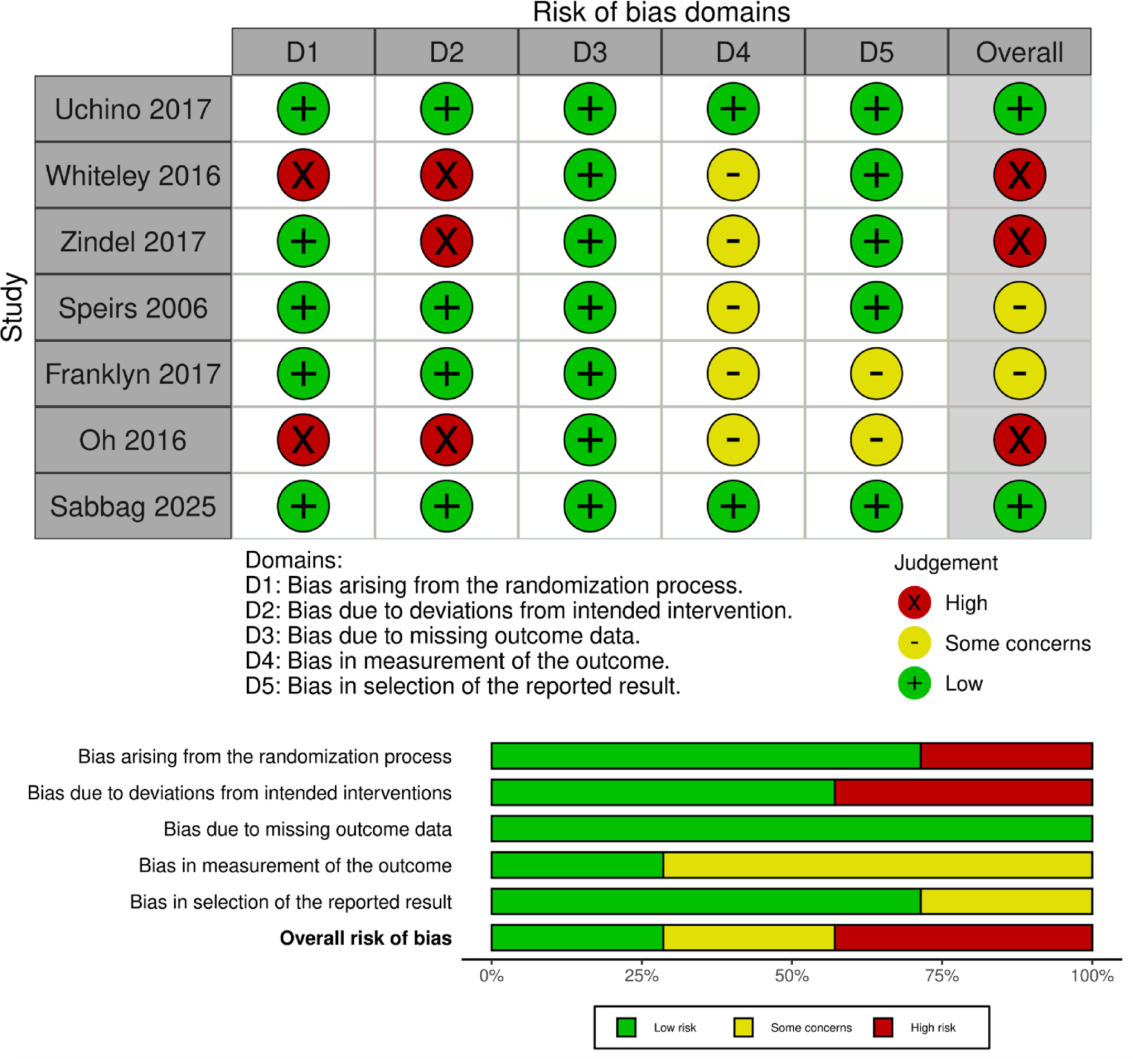
The bias-risk assessment diagram for the included studies

We utilized the Cochrane RoB2 tool to evaluate the methodological quality of the included studies. Three studies showed high risk of bias mainly due to deviations from intended intervention and concerns in the randomization process, while 2 studies showed low risk and 2 other studies showed some concerns due to bias in measurement of outcomes and selective reporting domains (Fig. 2).

### Outcomes

Analysis was done to compare the RR between the use of rod/bridge and no intervention in patients undergoing loop ileostomies/colostomies. Moreover, subgroup analysis was performed to investigate the outcomes at different time points.

#### Stoma retraction

**Fig. 3:**
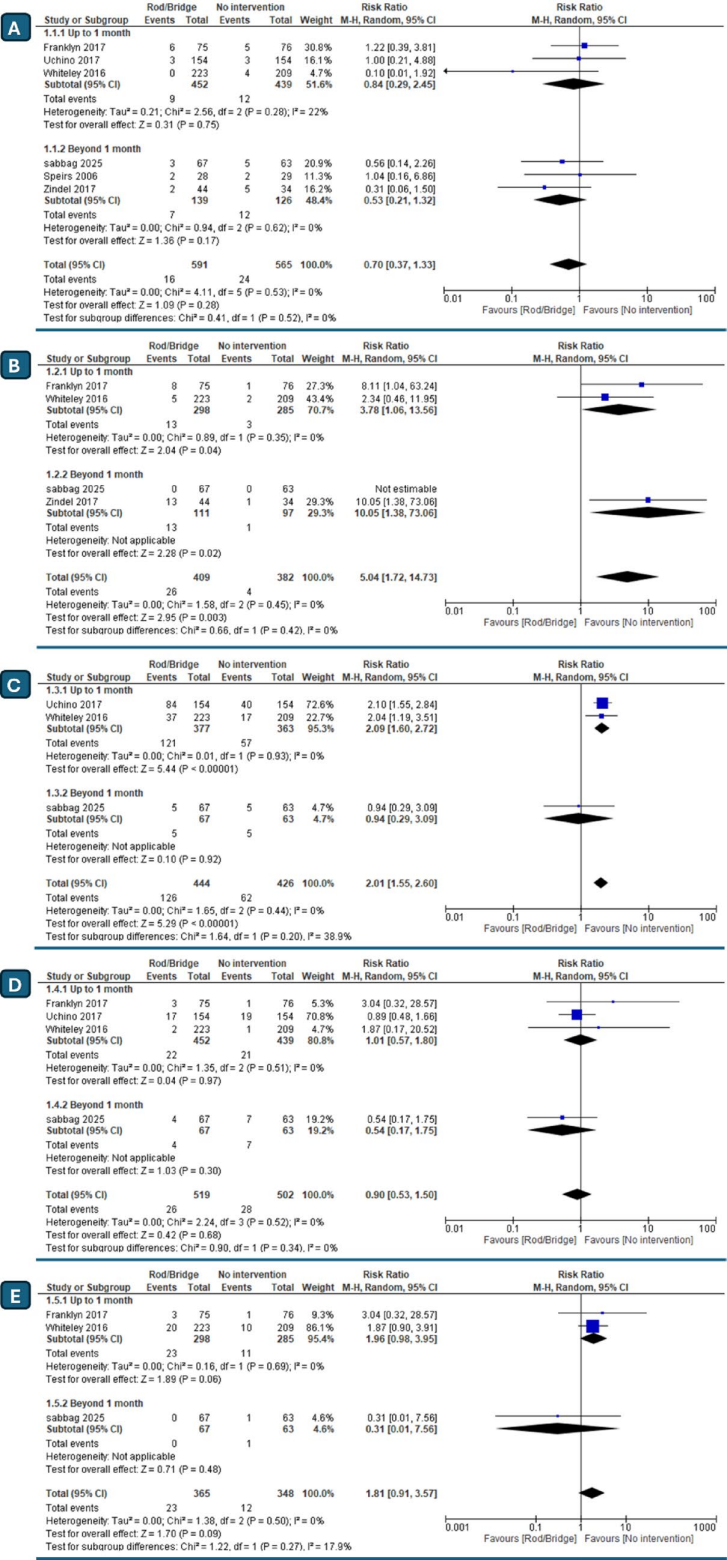
Forest plots comparing risk ratios (RR) of the use of bridge/rods in loop stomas; **A** stomal retraction, **B** stoma/skin necrosis, **C** dermatitis, **D** stomal/peristomal Infection, **E** mucocutaneous separation

Stoma retraction was assessed comparing rod/bridge and non-rod/bridge groups. Six studies including 1156 patients were included in this analysis revealing no statistically significant difference between either group (RR = 0.70, 95% CI [0.37 to 1.33], *P* = 0.28), with no heterogeneity detected (I^2^ = 0%, *P* = 0.45). Similarly, subgroup analysis showed no significant difference in either timepoint subgroups “up to 1 month” and “beyond one month” (RR = 0.84, 95% CI [0.29 to 2.45], *P* = 0.75), (RR = 0.53, 95% CI [0.21 to 1.32], *P* = 0.17) respectively. (Fig. 3A) TSA confirmed our findings with the α-spending adjusted CI (0.23 to 2.18) indicating a non-significant pooled RR. The final point on the cumulative z-curve didn’t pass the superiority monitoring boundary nor the conventional boundaries (False negative region) indicating a non-conclusive result. (Supplementary Fig. 1A) Moreover, the penalized Z-curve didn’t pass the conventional boundary of (z = 1.96). (Supplementary Fig. 1B) The required information size of 3061wasn’t reached.

#### Stoma/skin necrosis

Evaluating Stoma/Skin Necrosis, four studies with a total of 791 patients were included in our analysis. Surprisingly The overall results showed a statistically significant difference favoring the non- rod/bridge group over the rod/bridge group (RR = 5.04, 95% CI [1.72 to 14.73], *P* = 0.003) with no heterogeneity detected (I^2^ = 0%, *P* = 0.45). Similarly, subgroup analysis showed a statistically significant difference favoring the non- rod/bridge group in both subgroups “up to 1 month” and “beyond one month” (RR = 3.78, 95% CI [1.06 to 13.56], *P* = 0.04), (RR = 10.05, 95% CI [1.38 to 73.06], *P* = 0.02) respectively. (Fig. 3B). TSA confirmed our findings, as the penalized Z-curve passed the conventional boundary of (z = 1.96). (Fig. 6) However, the alpha Spending boundary was not renderable as the first information fraction exceeded 100% of RIS. (Supplementary Fig. 2).

#### Dermatitis

Assessing rates of peristomal dermatitis, three studies including 870 patients were included in our analysis. The overall results revealed a statistically significant difference favoring the non- rod/bridge group over the rod/bridge group (RR = 2.01, 95% CI [1.55 to 2.60], *P* < 0.00001), with no heterogeneity (I^2^ = 0%,* P* = 0.44). Similarly, Subgroup analysis showed a statistically significant difference in up to 1 month subgroup (RR = 2.09, 95% CI [1.60 to 2.72], *P* < 0.00001). However, the subgroup beyond 1 month revealed no statistically significant difference (RR = 0.94, 95% CI [0.29 to 3.09], *P* = 0.92). (Fig. 3C) TSA confirmed our findings as the penalized Z-curve passed the conventional boundary of (z = 1.96). (Supplementary Fig. 3) However, the alpha Spending boundary was not renderable as the first information fraction exceeded 100% of RIS.

#### Stoma site/peristomal infection

Evaluating Stoma Site/Peristomal Infection, four studies with 1021 patients were included in our analysis. The overall results revealed no statistically significant difference between groups (RR = 0.90, 95% CI [0.53 to 1.50], *P* = 0.68), with no heterogeneity (I^2^ = 0%, *P* = 0.52). Similarly, Subgroup analysis didn’t show any statistically significant difference in “up to 1 month” and “Beyond 1 month” subgroups (RR = 1.01, 95% CI [0.57 to 1.80], *P* = 0.97), (RR = 0.54, 95% CI [0.17 to 1.75], *P* = 0.30) respectively. (Fig. 3D) As for the TSA analysis, the penalized Z-curve didn’t pass the conventional boundary of (z = 1.96). However, the alpha spending Boundary was ignored due to too little information use (4.14%). (Supplementary Fig. 4).

#### Mucocutaneous separation

Mucocutaneous Separation risk evaluation included three studies encompassing 713 patients. The overall results revealed no statistically significant difference between either group (RR = 1.81, 95% CI [0.91 to 3.57], *P* = 0.09) with no heterogeneity (I^2^ = 0%, *P* = 0.50). Similarly, Subgroup analysis didn’t show any statistically significant difference in either “up to 1 month” and “Beyond 1 month” subgroups (RR = 1.96, 95% CI [0.98 to 3.95], *P* = 0.06), (RR = 0.31, 95% CI [0.01 to 7.56], *P* = 0.48) respectively. (Fig. 3E) TSA confirmed our findings with the α-spending adjusted CI (0.35 to 9.41) indicating a non-significant pooled RR. The final point on the cumulative z-curve didn’t pass the superiority monitoring boundary nor the conventional boundaries (False negative region) indicating a non-conclusive result. (Supplementary Fig. 5A) Moreover, the penalized Z-curve didn’t pass the conventional boundary of (z = 1.96). (Supplementary Fig. 5B) The required information size of 3367 wasn’t reached.

### Sensitivity analysis

To ensure the robustness of our evidence, we employed a leave one out sensitivity analysis in multiple scenarios, excluding one study in each scenario to ensure that the results weren’t dependent on a single study. Our findings revealed consistent stability, as the overall pooled significance remained unaffected across all outcomes. The only exception was in the analysis of Dermatitis, where the exclusion of either Uchino et al. or Whitely et al. resulted in a loss of statistical significance. (Supplementary Fig. 6–10).

### GRADE evaluation of evidence

We evaluated the certainty of evidence employing the GRADE approach, and the certainty of evidence ranged from moderate to low. Downgrades were mainly due to concerns about risk of bias and imprecision. Detailed summary of our findings with the GRADE assessment are shown in (Table 3) and (Supplementary Table 1).

**Table 3:** Summary findings and GRADE evaluation of RR (with vs without bridge)

Outcome	No. of patents (studies)	Relative effect (95% CI)	Anticipated absolute effects (95% CI)			*P*-value	Heterogeneity assessment *I* ^*2*^ * [P- value]*	Certainty of evidence
			No Bridge	Bridge	Difference			
Stomal retraction	1156 (6 studies)	RR 0.70 (0.37 to 1.33)	4.2%	3.0% (1.6 to 5.6)	1.3% fewer (2.7 fewer to 1.4 more)	*P* = 0.28	*I*^*2*^ = *0% [P* = *0.53]*	⨁⨁◯◯ **Low**
Stoma/skin necrosis	791 (4 studies)	RR 5.04 (1.72 to 14.73)	1.0%	5.3% (1.8 to 15.4)	4.2% more (0.8 more to 14.4 more)	*P* = 0.003*	*I*^*2*^ = *0% [P* = *0.45]*	⨁⨁⨁◯ **Moderate**
Dermatitis	870 (3 studies)	RR 2.01 (1.55 to 2.60)	14.6%	29.3% (22.6 to 37.8)	14.7% more (8 more to 23.3 more)	*P* < 0.00001*	*I*^*2*^ = *0% [P* = *0.44]*	⨁⨁⨁◯ **Moderate**
Stoma site/peristomal infection	1021 (4 studies)	RR 0.90 (0.53 to 1.50)	5.6%	5.0% (3 to 8.4)	0.6% fewer (2.6 fewer to 2.8 more)	*P* = 0.68	*I*^*2*^ = *0% [P* = *0.52]*	⨁⨁◯◯ **Low**
Mucocutaneous separation	713 (3 studies)	RR 1.81 (0.91 to 3.57)	3.4%	6.2% (3.1 to 12.3)	2.8% more (0.3 fewer to 8.9 more)	*P* = 0.09	*I*^*2*^ = *0% [P* = *0.50]*	⨁⨁◯◯ **Low**

Bold values indicate statistically significant results (*P* < 0.05)

## Discussion

This systematic review and meta-analysis examined the efficacy and safety of the use of rod or bridge during the formation of loop stomas. This review included five RCTs and a single observational study comprising a total of 1,239 patients who underwent either loop ileostomy (195 patients) or loop colostomy (1,044 patients). While the purpose of using a stoma rod is to provide support and prevent stoma retraction, its application has been counterintuitively linked to an increase in other complications as supported by recent studies [21, 22].

The analysis of stoma retraction, involving 1,156 patients from six studies, revealed no statistically significant difference between the two groups. Additionally, subgroup analysis according to the follow-up period (“up to 1 month” and “beyond 1 month”) demonstrated no significant difference between the groups. A meta-analysis by Du et al. [23], which included 1,131 patients with loop enterostomy from six studies, similarly showed that the incidence of stoma retraction in the rod group was not significantly lower than in the non-rod group (OR = 0.65, *P* = 0.23). Another meta-analysis conducted by Mohan et al. [24] identified five studies, with 561 patients undergoing stoma formation with a rod and 443 without; they found no significant difference in stoma retraction between the rod group (2.28%) and the no-rod group (3.45%) (OR = 0.7). Furthermore, Gachabayov et al. [25] identified three RCTs (392 patients) and reported stoma retraction rates of 3.1% in the rod group versus 4.5% in the no-rod group, a difference that was neither statistically nor clinically significant (OR = 0.60, *P* = 0.34).

In contrast, the studies consistently revealed a clear statistical difference concerning stoma or skin necrosis, with higher rates of necrosis reported in patients undergoing stoma or rod enterostomy. This significant disparity was observed in both subgroups. A meta-analysis by Du et al. [23] found that the incidence of stoma necrosis was significantly increased in the rod group (OR = 6.41, *P* = 0.0006). Similarly, Mohan et al. [24] also reported a significantly higher rate of stoma necrosis in the rod group (7%) compared to the no-rod group (1.15%) (OR = 5.58).

Our analysis of stoma site/peristomal infection, which included three studies with 870 patients, revealed a statistically significant difference favoring the non- rod/bridge group over the rod/bridge group (RR = 2.01, *P* < 0.00001. Du et al. [23] reported a significantly higher incidence of stoma retraction in the stoma support rod group (29.2% vs. 13.9%), with their meta-analysis indicating a statistically significant increase (OR = 2.93; *P* < 0.00001). Similarly, Mohan et al. [24] demonstrated a significantly increased risk of dermatitis in the rod group (29.86%) compared to the no-rod group (16%) (OR = 2.65).

For mucocutaneous separation, our risk evaluation, encompassing three studies with 713 patients, revealed no statistically significant difference between either group (RR = 1.81, P = 0.09). In contrast, Du et al. [23] reported a higher incidence of mucocutaneous separation in the rod group, with a statistically significant difference (OR = 2.14, *P* = 0.04). Mohan et al. [24], however, found no significant difference in mucocutaneous separation between the rod group (7.72%) and the no-rod group (3.86%) in two studies (OR = 2.06).

Overall, placing a stoma rod led to worse outcomes compared to not using it, with observed complications including swelling and inflammation around the stoma, tissue skin necrosis, skin damage due to moisture, abscesses, bleeding, and the separation of the stoma from the skin [26].

### Strengths and limitations

This systematic review constitutes a thorough meta-analysis that critically evaluates the application of rods and bridges in loop stomas, in strict accordance with PRISMA guidelines and the recommendations set forth by the Cochrane Collaboration. TSA was conducted to ensure the validity and conclusiveness of the meta-analytic results and minimize the risk of false positive, with parameters set for a type 1 error of 5% and type 2 error of 20% (80% statistical power), using a model variance-based heterogeneity correction. The uniformity in outcome metrics and the deployment of meta-analytic techniques enhance the credibility of the conclusions regarding the efficacy and safety associated with the use of rods and bridges. The GRADE framework was employed to evaluate the robustness of the evidence, with certainty levels ranging from moderate to low, primarily attributable to apprehensions regarding potential biases and imprecision.

Despite its strengths, this review has several limitations that may reduce the generalizability and strength of the findings. One of these shortcomings is the relatively limited number of the included studies. Furthermore, this review included one observational study, but a leave-one-out sensitivity analysis was performed to overcome this drawback. Additionally, the variations in patients’ characteristics across the studies contributed to the study limitation, particularly, the mix of patient cohorts undergoing different surgical procedures (ileostomy or colostomy) and presenting in varying clinical settings (elective or emergency) that introduced a source of variability that may have influenced the observed outcomes. Furthermore, the demographic features of the study populations were inadequately described or were heterogeneous, particularly regarding age and ethnic background. It was observed that the average BMI ranged from 19.5 to 26.1 kg/m^2^, which limits the applicability of the findings to individuals with obesity. The variation in age among the included studies, ranging from 41.9 ± 15.0 years to 64.6 ± 14.4 years, poses a further constraint. The follow-up periods in the included studies were also relatively brief, thereby limiting the ability to assess long-term efficacy and safety outcomes, such as evaluations extending beyond one month in the respective subgroups.

### Recommendations

The evidence gathered suggests that routinely using a rod or bridge during loop stoma formation doesn’t significantly reduce the occurrence of stoma retraction. However, their use is associated with a considerably higher risk of complications, including stoma and skin necrosis, as well as dermatitis. Given these findings, it’s advisable to initiate additional RCTs on this topic. These studies should be designed with extended follow-up durations to better understand the long-term effectiveness and safety of rods and bridges in loop stomas. There should be a particular focus on including a more representative and diverse patient group, especially those with elevated BMI and across wider age ranges.

## Conclusion

Based on the available evidence, routine rod/bridge use does not appear to reduce stoma retraction and may be associated with an increased risk of significant complications, such as necrosis and dermatitis. Given these findings, the routine use of this technique does not appear to be justified, however the small number of the studies included limit the generalizability of these results. Trial sequential analysis indicated that future high-quality RCTs with diverse and larger populations are recommended to obtain more rigorous evidence.

## Supplementary Information

Below is the link to the electronic supplementary material.

Supplementary Material 1

## Data Availability

The datasets used and/or analyzed during the current study are available from the corresponding author on reasonable request.
