# Supplementary material for: Supporting or complicating? The role of rods and bridges in loop stomas: a comprehensive systematic review and meta-analysis with GRADE evaluation and trial sequential analysis
Source: Updates Surg. 2025 Dec 2;78(2):637–47. doi: 10.1007/s13304-025-02455-z (PMC13212795; doi:10.1007/s13304-025-02455-z)
Supplement: Supplementary file 1 — Supplementary Material 1 [file 13304_2025_2455_MOESM1_ESM.docx]

**Supplementary Table. 1** Details of GRADE of Evidence

| **Outcome** | **Certainty of Evidence** | | | | | | |
| --- | --- | --- | --- | --- | --- | --- | --- |
|  | **Risk of bias** | **Indirectness** | **Inconsistency** | **Imprecision** | **Publication bias** | **Other considerations** | **Overall certainty of evidence** |
| **Stomal retraction** | Downgraded ^a^ | No | No | Downgraded ^b^ | N/A | No | **⨁⨁◯◯ Low^a,b^** |
| **Stoma/Skin Necrosis** | Downgraded ^a^ | No | No | No | N/A | No | **⨁⨁⨁◯ Moderate^a^** |
| **Dermatitis** | Downgraded ^a^ | No | No | No | N/A | No | **⨁⨁⨁◯ Moderate^a^** |
| **Stoma Site/Peristomal Infection** | Downgraded ^a^ | No | No | Downgraded ^b^ | N/A | No | **⨁⨁◯◯ Low^a,b^** |
| **Mucocutaneous Separation** | Downgraded ^a^ | No | No | Downgraded ^b^ | N/A | No | **⨁⨁◯◯ Low^a,b^** |

Explanations:

a. This outcome was rated down for risk of bias as the majority of included studies showed a High overall risk of bias mainly due to bias due to deviation from intended intervention and randomization domains while other studies showed some concerns due to bias in measurement of outcomes and in selection of reported results domains.

b. Downgraded for imprecision owing to the wide 95% CI which includes clinically important differences, moreover TSA revealed that RIS wasn’t reached

**
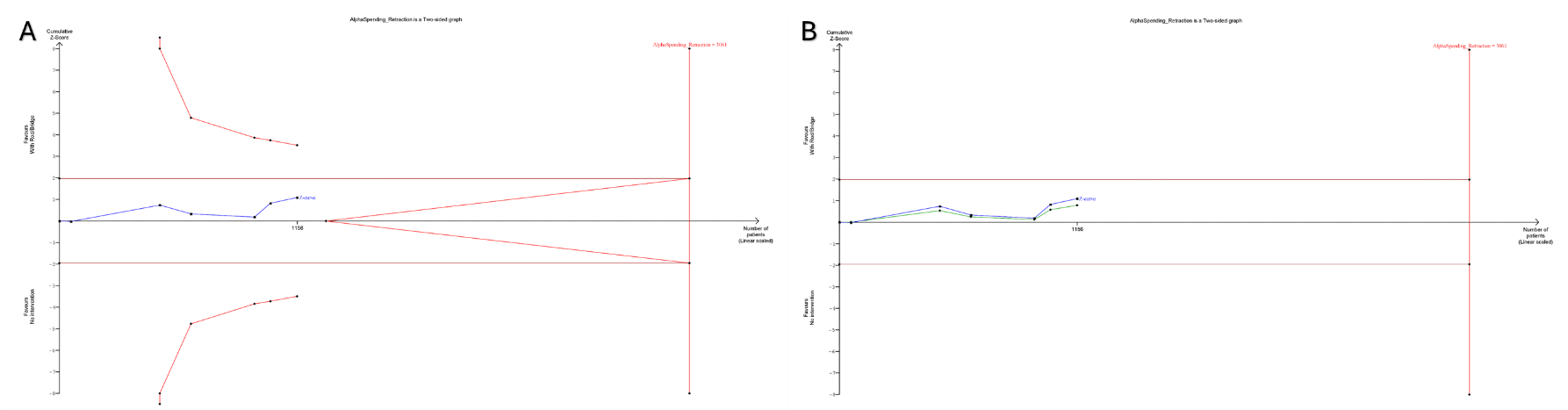
**

**Supplementary Figure. 1** TSA on Relative Risks (RR) for Stoma retraction

**Fig. 1A** Stoma retraction cumulative Z-curve not passing the superiority boundary or the conventional boundary (False negative).

**Fig. 1B** Stoma retraction penalized Z-curve not passing the conventional boundary.


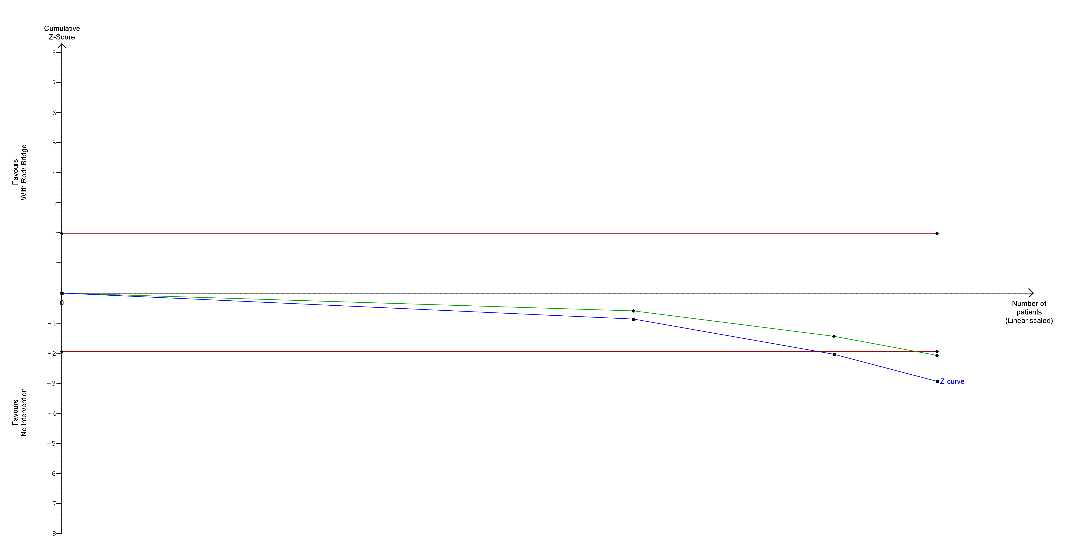


**Supplementary Figure. 2** TSA on Relative Risks (RR) for Stoma/Skin Necrosis

(Stoma Necrosis penalized Z-curve passing the conventional boundary)


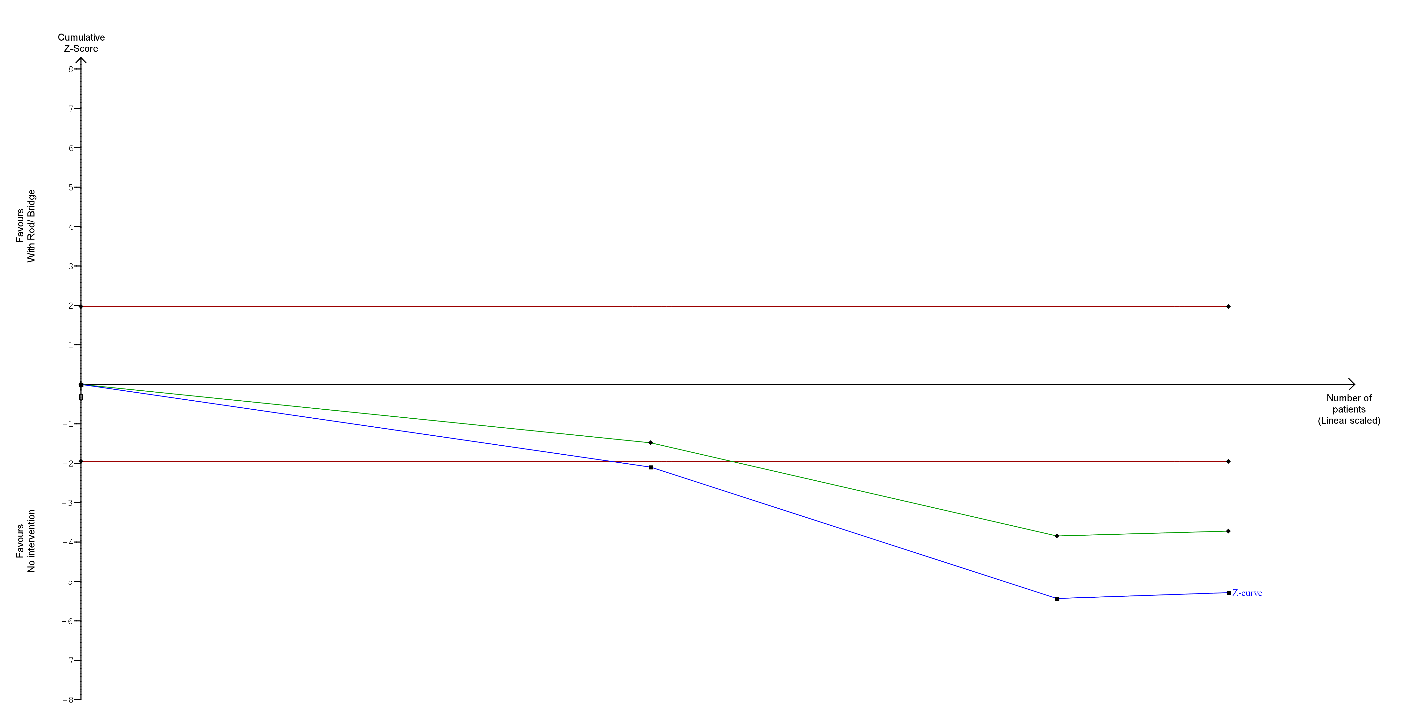


**Supplementary Figure. 3** TSA on Relative Risks (RR) for Dermatitis

(Dermatitis penalized Z-curve passing the conventional boundary)


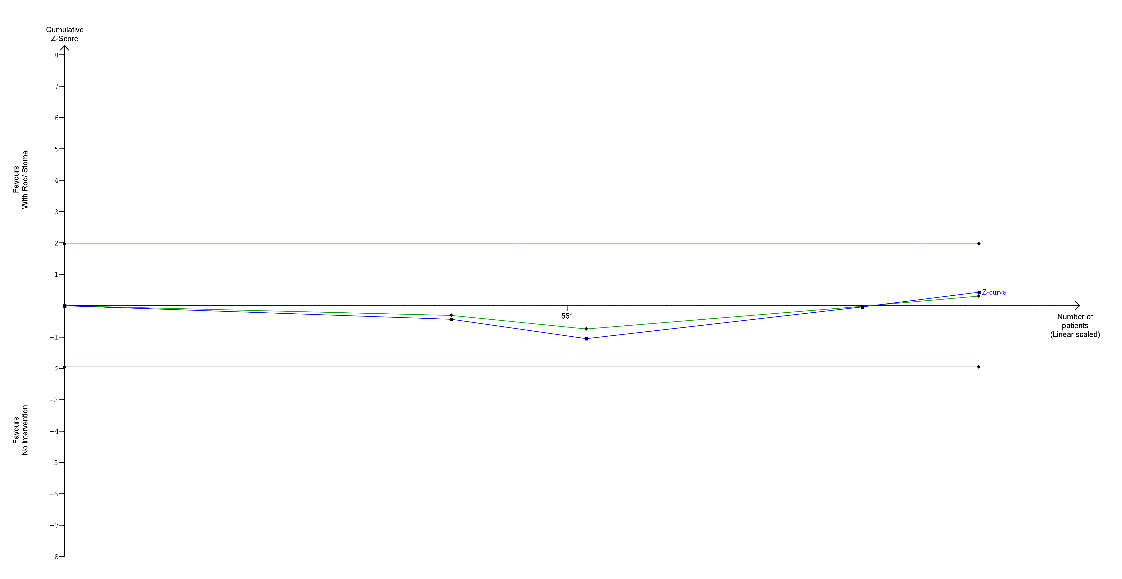


**Supplementary Figure. 4** TSA on Relative Risks (RR) for Stomal/Peristomal Infection

(Infection penalized Z-curve not passing the conventional boundary)


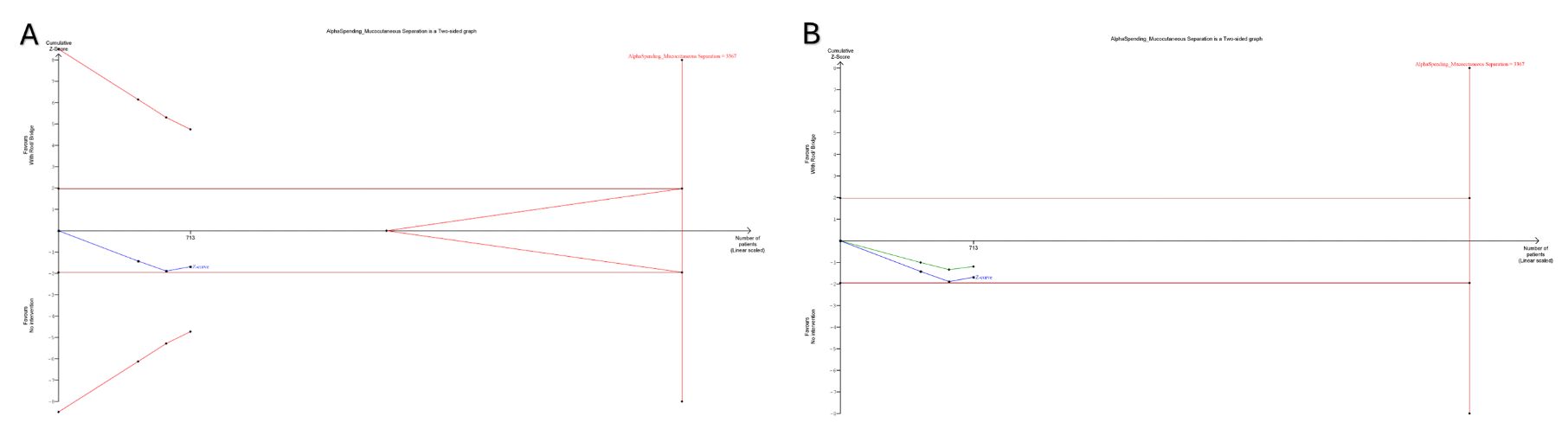


**Supplementary Figure. 5** TSA on Relative Risks (RR) for Mucocutaneous separation

**(Fig. 5A)** Mucocutaneous separation cumulative Z-curve not passing the superiority boundary or the conventional boundary (False negative).

**(Fig. 5B)** Stoma retraction penalized Z-curve not passing the conventional boundary.

**
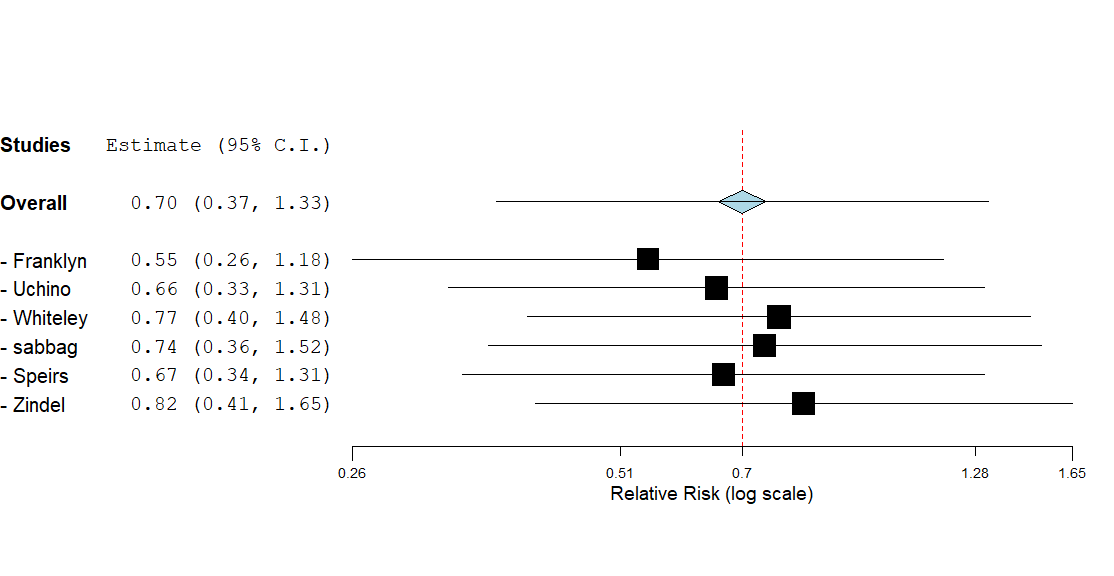
**

**Supplementary Figure. 6** Leave one out sensitivity analysis for Stoma retraction


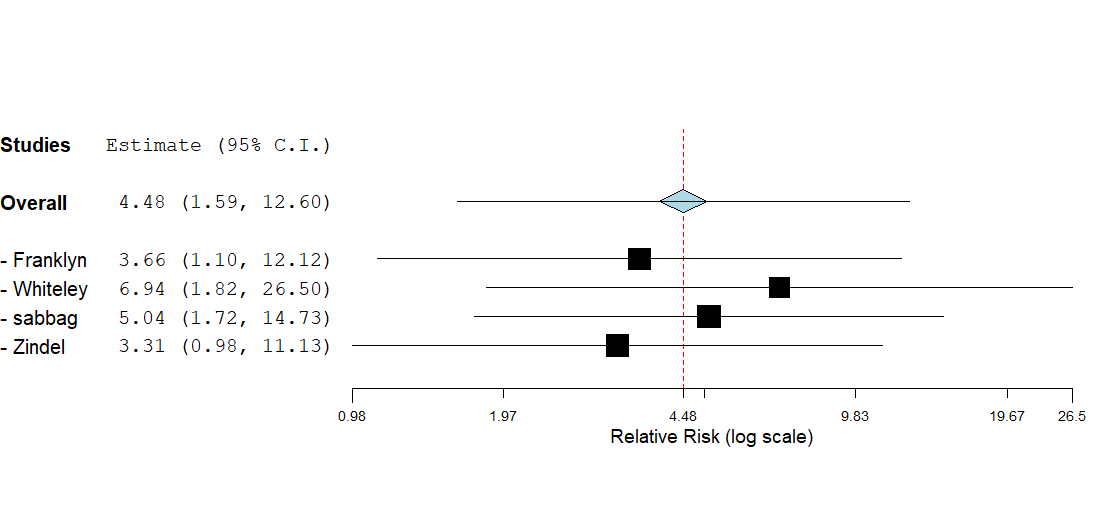


**Supplementary Figure. 7** Leave one out sensitivity analysis for Stoma/Skin Necrosis


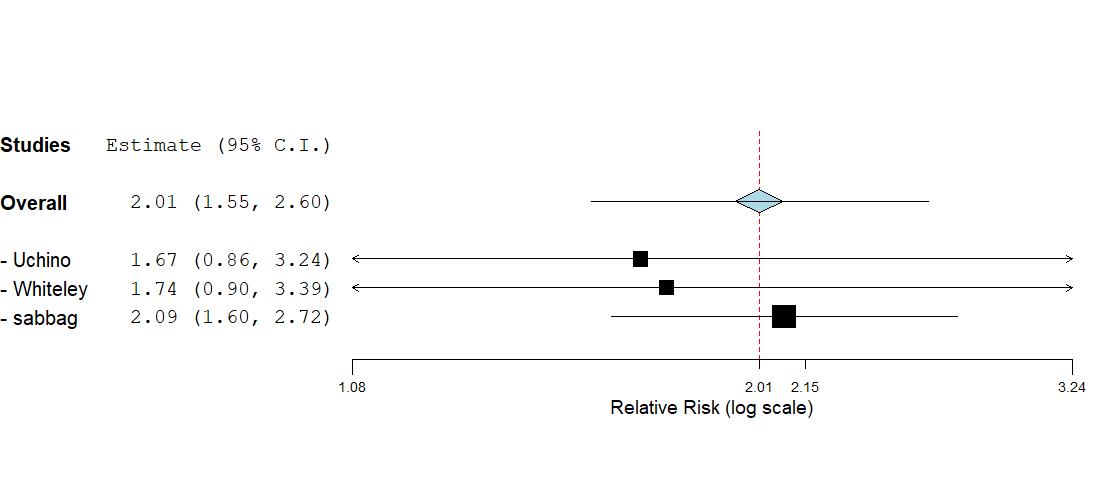


**Supplementary Figure. 8** Leave one out sensitivity analysis for Dermatitis


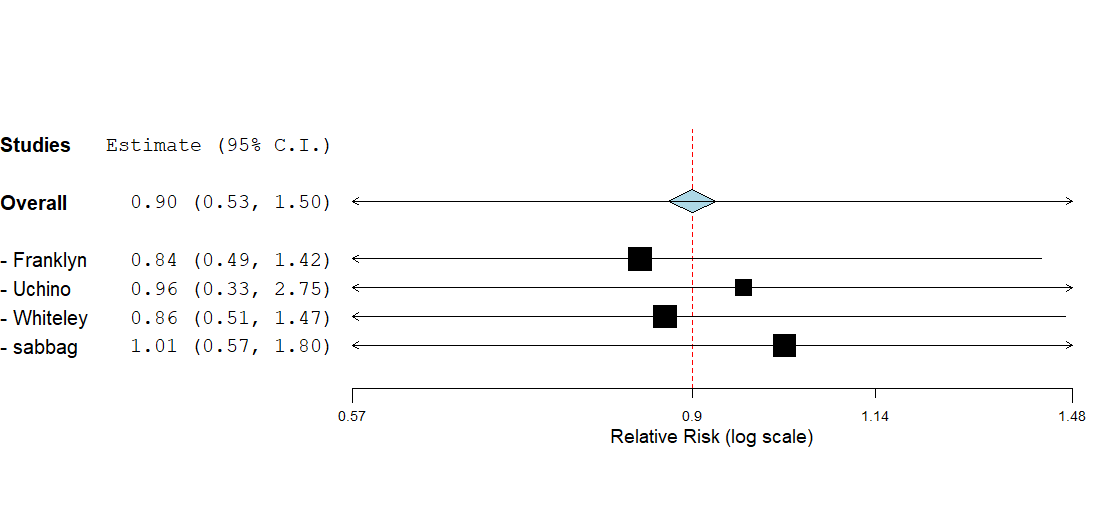


**Supplementary Figure. 9** Leave one out sensitivity analysis for Stoma Site/Peristomal Infection.


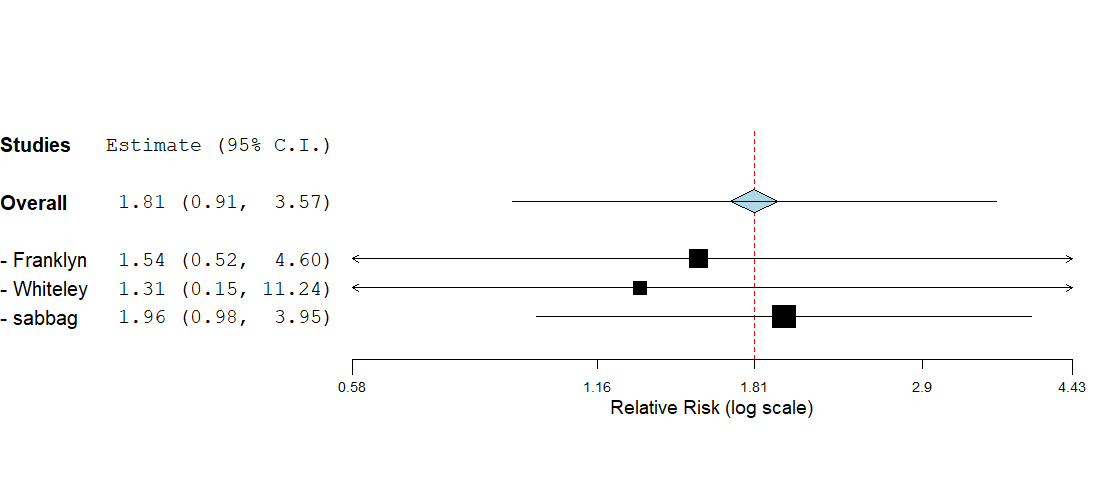


**Supplementary Figure. 10** Leave one out sensitivity analysis for Mucocutaneous separation.
